# Measurement precision at the cut score in medical multiple choice exams: Theory matters

**DOI:** 10.1007/s40037-020-00586-0

**Published:** 2020-05-28

**Authors:** Felicitas-Maria Lahner, Stefan Schauber, Andrea Carolin Lörwald, Roger Kropf, Sissel Guttormsen, Martin R. Fischer, Sören Huwendiek

**Affiliations:** 1 Institute for Medical Education, University of Bern, Bern, Switzerland; 2 Department of Health Professions, University of Applied Sciences, Bern, Switzerland; 3 Centre for Educational Measurement at the University of Oslo (CEMO) and Centre for Health Sciences Education, University of Oslo, Oslo, Norway; 4 Faculty of Medicine, University of Zurich, Zurich, Switzerland; 5 Institute for Medical Education, University Hospital, LMU Munich, Munich, Germany

**Keywords:** Multiple choice exams, Measurement precision, Reliability, Conditional reliability

## Abstract

**Introduction:**

In high-stakes assessment, the measurement precision of pass-fail decisions is of great importance. A concept for analyzing the measurement precision at the cut score is conditional reliability, which describes measurement precision for every score achieved in an exam. We compared conditional reliabilities in Classical Test Theory (CTT) and Item Response Theory (IRT) with a special focus on the cut score and potential factors influencing conditional reliability at the cut score.

**Methods:**

We analyzed 32 multiple-choice exams from three Swiss medical schools comparing conditional reliability at the cut score in IRT and CCT. Additionally, we analyzed potential influencing factors such as the range of examinees’ performance, year of study, and number of items using multiple regression.

**Results:**

In CTT, conditional reliability was highest for very low and very high scores, whereas examinees with medium scores showed low conditional reliabilities. In IRT, the maximum conditional reliability was in the middle of the scale. Therefore, conditional reliability at the cut score was significantly higher in IRT compared with CTT. It was influenced by the range of examinees’ performance and number of items. This influence was more pronounced in CTT.

**Discussion:**

We found that conditional reliability shows inverse distributions and conclusions regarding the measurement precision at the cut score depending on the theory used. As the use of IRT seems to be more appropriate for criterion-oriented standard setting in the framework of competency-based medical education, our findings might have practical implications for the design and quality assurance of medical education assessments.

## Introduction

This study examines the nature of measurement precision at the cut score as estimated according to Classical Test Theory (CTT) and Item Response Theory (IRT). In the following, we will begin by describing why it is important to determine the measurement precision at the cut score, and by introducing the concept of conditional reliability and its manifestation in CTT and IRT. We will then describe factors influencing conditional reliability and formulate the research questions.

In medical education, high-stakes assessment decisions can have far-reaching consequences both for students and for society. If competent students fail an exam, this hinders their career progress, and if incompetent students pass an exam, it can put patients at risk. Hence, making defensible pass-fail decisions and providing arguments for the trustworthiness of these decisions is of vital importance in high-stakes medical education assessment [1, 2]. Measurement precision is an important issue in this context [3], and describes the extent to which an assessment is free of random error, meaning that scores are consistent across different observations [3]. Students with sufficient ability who have passed an exam should also pass another, similar exam, whereas students with insufficient ability should consistently fail both exams. Therefore, whenever a test score from an assessment is used to make a decision about a specific examinee, such as passing or failing an exam, it is vital to achieve an adequate threshold of measurement precision [4]. An index that is commonly used for reporting measurement precision is Cronbach’s alpha [5], which provides one global index of measurement precision for an exam. However, this index is not appropriate for dichotomous decisions such as passing or failing a student [6].

According to psychometric theory, the precision with which test scores are measured is assumed to vary across score levels [7–11]. Therefore, when the passing and failing of students is of great importance, an index is required that reports measurement precision at the point that matters, which in most cases is the cut score. In this regard, the concepts of conditional reliability and conditional standard error of measurement (cSEM) can offer relevant information [4, 12]. The cSEM provides an estimate of the amount of measurement error for every individual test score. As the cSEM is interpreted in relation to the scale employed, it cannot be used to compare measurement precision between exams [12] (i.e. the interpretation of the same cSEM would differ according to whether exam scores range from 0–10 or from 0–100). Raju and colleagues [12] proposed a standardization of the cSEM, an index of conditional reliability, which reports a reliability coefficient at the level of individual test scores and allows for easier interpretation and comparisons across measurement instruments, e.g. across different exams.

Conditional reliability, as proposed by Raju et al. [12], is defined as a function of, first, the between-person variance and, second, the examinee-level standard error of measurement. In other words, it is defined as the standardized conditional standard error of measurement: $cRel=\frac{\sigma _{x}^{2}-\mathrm{cSEM}^{2}}{\sigma _{x}^{2}}$. Interestingly, Raju et al. [12] showed that estimates of conditional reliability vary between IRT and CTT, sometimes drastically. In CTT, conditional reliability was found to be high for extreme scores and low for medium scores. The opposite was shown for IRT, where conditional reliability was low for very high and low scores and high for medium scores. These differences can be explained by the two theories’ different conceptions of measurement error on the level of the individual test scores (for details see Mellenbergh [13] or DeMars [14]). As both IRT and CTT are used to analyze medical education assessment, this observation has some important practical implications. When analyzing the measurement precision at the cut score, conclusions about whether or not high-stakes decisions are made with an adequate level of precision can diverge substantially depending on the psychometric framework employed. Hence, in this context, opting for one of the psychometric frameworks based on theoretical considerations really matters.

Therefore, it is necessary to understand how conditional reliability may be influenced in both theories in the specific context of high-stakes medical education assessment. There are two crucial factors that affect estimates of conditional reliability, which are typical characteristics of assessment in medical education.

First, in both theories, between-person variance affects estimates of conditional reliability: Estimates of between-person variance are used to standardize the cSEM. In the case of Raju et al. [12], scores covered the whole possible range from 0% to 100% correct responses. In the context of high-stakes assessment in medical education, however, it has long been noted that between-person variation is often rather low. Often, only as much as 2% of the total variance is attributable to stable between-person differences [15]. The range of examinees’ performance in high-stakes exams is typically restricted, as there are hardly any exams where scores range from 0% correct to 100% correct answers. This restriction of variance might be enhanced further by the very nature of assessment; that is, poor-performing students are usually forced to drop out of medical school, further restricting the variation in medical students’ ability levels, especially in the final years of medical school. Thus, both restriction of range and the year of study might affect estimates of conditional reliability.

Second, in both CTT and IRT, the length of a test (i.e., the number of items included) affects measurement precision. By and large, longer tests measure more precisely than shorter tests. However, a crucial difference between the two theories is that in IRT, psychometric characteristics of the items (i.e., difficulty, discrimination, etc.) determine at which score level these scores are measured and with which degree of precision [14, 16]. Tests in high-stakes medical education assessment can be quite long, with up to 300 items for licensing examinations [17]. To date, it remains unclear to what extent the test length affects estimates of conditional reliability differently in CTT and IRT.

Understanding these influences and how they interact with the employed psychometric framework is a prerequisite for the regular use of conditional reliability in medical education assessment. So far, no research has compared conditional reliability in CCT and IRT while taking into account relevant real-life conditions of medical education assessment. In this study, we analyze conditional reliability in both CTT and IRT in the context of high-stakes medical education assessment and their relation to the aforementioned influencing factors. Our first research question is whether we can replicate previous findings regarding the areas with high and low precision in medical education assessment (i.e. high conditional reliability for medium ability levels in IRT and high conditional reliability for high and low ability levels in CTT). As the precision with which scores around the cut score are measured is of great importance [16], the second research question is how the conditional reliability at the cut score compares between the two theories. The third research question is whether the aforementioned relevant factors, namely range of examinees’ performance, year of study and number of items, influence measurement precision at the cut score differently in CTT and IRT.

We believe that these research questions are highly relevant to improve the understanding of measurement precision at the cut score in high-stakes medical education assessment. Results may have practical implications for the quality assurance of pass-fail decisions and even for assessment design.

## Methods

### Sample

For this study, we analyzed 32 high-stakes medical end-of-term exams from three Swiss medical schools conducted in 2016. Our sample covered exams ranging from the first to the fifth year of study. End-of-term exams cover the entire content taught in that term and are used to decide whether a candidate is allowed to pass the term and to continue her or his studies. All included exams were constructed according to the blueprints of the programs and terms, which are all based on the Swiss Catalogue of Learning Objectives [18, 19], and met high-quality standards, e.g. careful item review and revision according to the standards set by Haladyna, Downing [20] and Case and Swanson [21].

The mean number of examinees per exam was 264 (SD = 83; min = 146; max = 378). All exams were multiple-choice exams comprising single-best answer (Type A) items and multiple true-false (MTF) items. The mean number of items per exam was 103 (SD = 428; min = 59; max = 150). On average 30.60% of the items were MTF items (SD = 8.00% min = 18.97%, max = 53.33%). Type A items included five answer options, and MTF items included four answer options. Type A items were scored with a full point when answered correctly; otherwise, examinees received no points. MTF items were scored using a partial credit scoring algorithm [22, 23]. For these items, examinees received half a point if more than half of true/false ratings of an item were marked correctly and one point if all were marked correctly. Otherwise, they received no points for the item. Items eliminated in post-hoc review were excluded from analyses (1.5 items per exam on average). Item difficulty covered the whole range, from easy to difficult items (min = 0.018, mean = 0.69, max = 1).

The standard setting of all exams was content-based [24]. Cut scores ranged from 47.5% to 70% of the maximum points, with a mean at 56.6% (SD = 4.7%).

### Calculation of conditional reliability

We calculated conditional reliabilities for every exam in both IRT and CTT [12]. In both theories, conditional reliability is a standardization of the cSEM at the score variance (σ_x_^2^). Conditional reliability is defined as:

$$cRel=\frac{\sigma _{x}^{2}-\text{cSEM}}{\sigma _{x}^{2}}$$

To calculate the cSEM in CTT, we used the binominal error model [7, 8]. According to this model, the cSEM is defined as follows:

$$cSEM=\sqrt{\frac{X\left(k-X\right)}{k-1}}$$

where X is the score of an exam and k is the number of items.

In IRT the squared cSEM is inversely equal to the test information function (I_s_) [12]. The cSEM is calculated as follows:

$$cSEM=\sqrt{\frac{1}{I_{s}}}$$

To calculate conditional reliability in IRT, we used a one-parameter logistic (1-PL) IRT model for partial credit scoring. In this model, every score on the theta scale corresponds to only one test score on the sum score “scale”. This correspondence is useful for judging the differences between the two approaches. For estimating theta scores, we used the weighted likelihood estimator [25].

Local independence is a prerequisite for applying a 1-PL model. For testing local independence, we used the Q3 statistic [26, 27]. Mean Q3 value was 0.06 (min = 0.05, max = 0.07), indicating that the data are locally independent.

The 1‑PL model showed an acceptable fit for the data. The mean SRMR (standardized root mean square residual) was 0.06 (min = 0.05, max = 0.08), and the mean SRMSR (standardized root mean square root of squared residual) was 0.08 (min = 0.06 max = 0.14). We also calculated Infit and Outfit for the items in the included exams. On average 4% (min = 0.00%, max = 16.67%) of the items in an exam did not fit with regard to the Infit. Regarding the Outfit, on average 12.57% (min = 0.00%, max = 40%) of the items did not fit. Items that did not fit with regard to the Outfit were mostly easy items with low discrimination indices. (Tab. 1).

**Table 1:** Fit indices for each exam

		Infit				Outfit				SRMR	SRMSR
Exam	Q3	Min	Max	Mean	% not fitting items	Min	Max	Mean	% not fitting items		
1	0.06	0.88	1.26	1.02	6.67	0.40	1.57	1.03	10.67	0.067	0.084
2	0.07	0.85	1.36	1.01	2.00	0.00	2.04	0.98	13.33	Inf	Inf
3	0.06	0.90	1.34	1.03	3.47	0.56	1.92	1.05	6.25	0.071	0.089
4	0.06	0.00	1.24	1.03	4.05	0.00	1.47	1.05	8.11	0.068	0.085
5	0.07	0.90	1.26	1.02	1.68	0.36	1.87	1.03	3.36	0.078	0.098
6	0.07	0.89	1.21	1.02	1.67	0.00	1.33	1.01	1.67	0.073	0.092
7	0.07	0.90	1.18	1.02	0.00	0.48	1.57	1.04	8.55	0.072	0.089
8	0.07	0.88	1.21	1.01	3.33	0.56	1.53	1.00	6.67	0.078	0.098
9	0.07	0.92	1.13	1.01	0.00	0.00	1.67	1.00	1.68	0.069	0.087
10	0.05	0.91	1.22	1.02	6.78	0.79	1.27	1.03	23.73	0.060	0.076
11	0.05	0.90	1.20	1.02	6.78	0.54	1.48	1.03	20.34	0.061	0.077
12	0.05	0.90	1.27	1.01	6.78	0.64	1.36	1.02	16.95	0.067	0.084
13	0.06	0.88	1.18	1.01	3.33	0.52	1.33	0.99	15.00	0.068	0.087
14	0.05	0.90	1.22	1.02	3.70	0.65	2.16	1.03	14.81	0.059	0.074
15	0.05	0.87	1.30	1.03	5.13	0.57	2.17	1.06	23.08	0.065	0.081
16	0.07	0.94	1.14	1.02	0.00	0.67	1.94	1.04	1.72	0.067	0.083
17	0.06	0.93	1.08	1.01	0.00	0.85	1.30	1.01	0.00	0.066	0.082
18	0.06	1.00	1.00	1.00	0.00	1.00	1.00	1.00	0.00	0.063	0.081
19	0.05	0.76	1.31	1.00	11.67	0.22	1.57	0.95	40.00	0.074	0.093
20	0.05	0.89	1.28	1.02	10.00	0.74	1.66	1.04	23.33	0.063	0.079
21	0.07	0.91	1.39	1.02	6.67	0.77	1.51	1.01	26.67	0.079	0.140
22	0.05	0.83	1.40	1.03	16.67	0.52	1.57	1.04	33.33	0.065	0.082
23	0.05	0.90	1.23	1.02	5.56	0.74	1.48	1.02	21.11	0.058	0.073
24	0.06	0.92	1.13	1.01	2.22	0.71	1.51	1.02	2.22	0.066	0.121
25	0.05	0.91	1.17	1.02	2.02	0.62	1.90	1.03	10.10	0.053	0.066
26	0.05	0.86	1.22	1.02	9.09	0.46	1.41	1.01	19.19	0.057	0.072
27	0.05	0.84	1.24	1.01	0.00	0.38	2.46	1.03	22.00	0.062	0.078
28	0.05	0.90	1.28	1.01	6.12	0.40	1.42	1.01	13.27	0.057	0.072
29	0.05	0.94	1.17	1.03	0.00	0.00	1.58	1.04	5.04	0.056	0.070
30	0.05	0.94	1.10	1.01	0.00	0.56	1.23	1.00	0.00	0.056	0.070
31	0.05	0.95	1.12	1.01	1.72	0.68	2.03	1.01	5.17	0.055	0.069
32	0.05	0.93	1.14	1.02	1.65	0.69	1.21	1.02	4.96	0.055	0.069

Conditional reliability at the cut score as well as maximum and average conditional reliability were calculated. As an index of global reliability, the Cronbach’s alpha (in CTT) and separation index (in IRT) were calculated for each exam.

### Influencing variables

The three influencing variables mentioned above were included in our analyses in order to test whether they relate to differences in conditional reliability at the cut score between exams: (1) range of examinees’ performance, (2) year of study, (3) number of items. As an index for the range of examinees’ performance, we used the difference between the maximum and minimum score in an exam. To enable comparison between the exams, examinees’ performance was calculated in percent for all analyses. As we used anonymized data, we were not able to include examinee-specific factors.

### Control variables

Exams included in this study are from three different medical schools and contain both Type A and MTF items. The amount of MTF items may influence the test information and thereby conditional reliability. Therefore, we included both medical schools and the percentage of MTF items in the exams as control variables in the regression analyses.

### Statistical analyses

To compare the conditional reliability at the cut score in IRT and CTT and to analyze influencing factors, we used analyses of variance (ANOVA) as well as regression analyses. As an index of effect size, we report partial eta^2^ and standardized beta. The level of significance was set at *p* < 0.5. All analyses were conducted using R (version 3.2.0) [28]. To estimate the 1PL IRT model, we used the R package “TAM” [29] and for graphics, we used the R package “ggplot2” [30].

## Results

### Conditional reliability

**Fig. 1:**
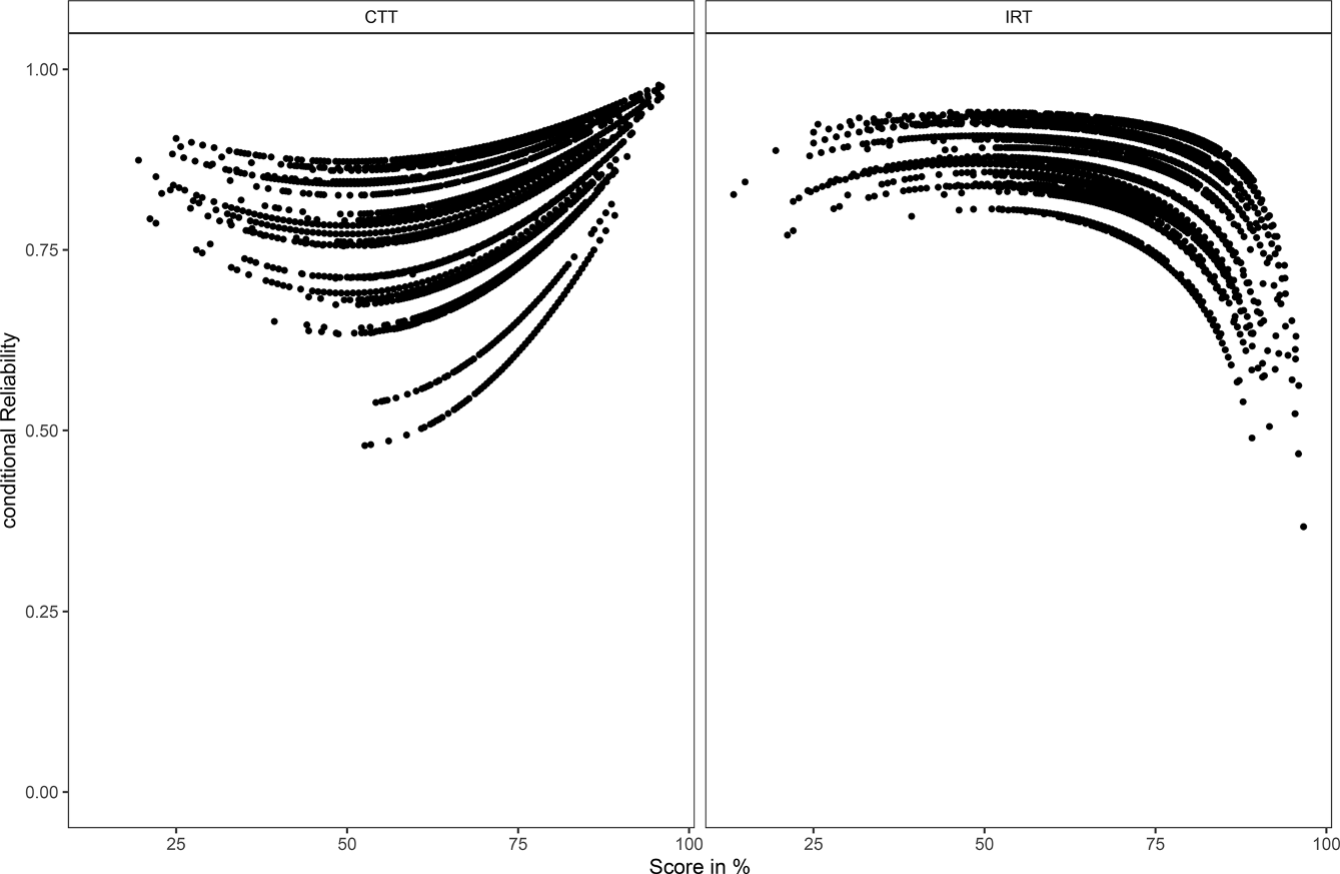
Mean conditional reliability and standard deviation in classical test theory (CTT) and item response theory (IRT) calculated over 32 exams

Our first research question was whether we can replicate previous findings regarding the areas with high and low precision in CTT and IRT, employing data obtained from high-stakes assessment in medical education. We indeed found high conditional reliability in CTT for the high and low scores, with a maximum of 0.96 for examinees with 95% correct answers. For medium scores, with 50% and 60% correct answers, we found conditional reliability to be lower, with a minimum of 0.74. In IRT, we found conditional reliability for low and medium levels to be above 0.75, with a maximum of 0.89 for scores around 50% correct. For the very high scores, conditional reliability was lower, with a minimum of 0.58 for 95% correct answers. The graphical display can be found in Fig. 1. The grand means for the separation index and Cronbach’s alpha were identical (alpha = 0.85, separation index = 0.85).

### Conditional reliability at the cut score

The second research question was how the reliability at the cut score compares between the two theories. In CTT, conditional reliability at the cut score ranged from 0.52 to 0.96 (mean = 0.75, SD = 0.09). In IRT, conditional reliability at the cut score ranged from 0.79 to 0.94 (mean = 0.88, SD = 0.04) and was significantly higher (F(1/31) = 162.13, *p* < 0.05, eta^2^ = 0.46).

### Influencing variables

The third research question was whether homogeneity of examinees’ performance, year of study and number of items influence measurement precision at the cut score differently in CTT and IRT. Due to multicollinearity, we had to exclude the year of study from the analyses. We also included the two control variables medical school and percentage of MTF items in this analysis. We found a significant regression equation (F(9/54) = 36.2, *p* < 0.05) with an R^2^ of 0.86. Regression coefficients can be found in Tab. 2. In the following, we will describe the results of each variable.

**Table 2:** Regression analyses to analyze the influence of the used theory, the number of items, the range of examinees’ performance, medical school, percentage of MTF items and interactions between the respective theory and the five other variables on conditional reliability at the cut score using, displaying the unstandardized beta (B), the standard error for the unstandardized beta (SE(B)), the standardized beta (β), the t‑test statistic (t), and the probability value (*p*)

Variable	B	SE (B)	β	t	*p*
Intercept	−0.542	0.227	0.000	0.000	<0.05
Theory	0.533	0.144	0.677	13.190	<0.05
Range of examinees’ performance	0.011	0.002	0.753	10.503	<0.05
Number of items	0.003	0.001	0.521	6.531	<0.05
Medical school	0.020	0.032	0.078	0.901	0.37
Percentage of MTF items	0.471	0.316	0.130	1.747	0.09
Theory* Range of examinees’ performance	−0.004	0.001	−0.282	−3.904	<0.05
Theory* Number of items	−0.001	0.001	−0.150	−1.868	0.07
Theory* Medical school	−0.007	0.020	−0.032	−0.362	0.72
Theory* Percentage of MTF items	−0.197	0.200	−0.074	−0.988	0.33

### Range of examinees’ performance

Across exams, performances ranged from a minimum of 13.33% to a maximum of 98.33%. Within exams, the highest range of examinees’ performance was 83.33%, with scores varying from 13.33% to 96.67% correct. The smallest range of examinees’ scores was 34.45%, with scores varying from 54.20% to 88.66% correct. Regarding conditional reliability at the cut score, we found a significant influence of the range of examinees’ performance (*B* = 0.011, *β* = 0.75, *p* < 0.05) as well as a significant interaction between the range of performances in the exams and the theory used (*B* = −0.004, *β* = −0.28, *p* < 0.05). The range restriction influenced conditional reliability, leading to lower reliability in both CTT and IRT. The significant interaction shows that the restriction of range has a higher impact on conditional reliability at the cut score in CTT. For the exam with the largest range in test scores (range = 83.33%), conditional reliability at the cut score was 0.96 in CTT and 0.93 in IRT. The exam with the lowest range in test scores (range = 34.45%) had a conditional reliability at the cut score of 0.56 in CTT and 0.83 in IRT.

### Year of study

We included exams from all five years of study. In CTT, conditional reliability at the cut score decreased from a mean of 0.81 in the first-year exams to 0.56 in the fifth-year exams. In IRT, conditional reliability at the cut score decreased from a mean of 0.90 in the first-year exams to 0.83 in the fifth-year exams. We did not include year of study in the regression analyses due to multicollinearity. Year of study showed a high correlation with range of examinees’ performance (*r* = −0.78, *p* < 0.05) and a medium correlation with number of items ( *r* = 0.48, *p* < 0.05) (Tab. 3). Therefore, results would be similar to those of the range of examinees’ performance.

**Table 3:** Correlations between influencing variables (*p < 0.05)

	Year of study	Number of items
Number of items	0.48*	
Range of examinees’ performance	−0.78*	−0.67*

### Number of items

The number of items per exam ranged between 59 and 150 (mean = 103). Regarding conditional reliability at the cut score, we found a significant effect of the number of items (*B* = 0.003, *β* = 0.452, *p* < 0.05) but no significant interaction with the used theory (*B* = −0.001, *β* = −0.15, *p* = 0.07). A higher number of items led to higher conditional reliability. The exam with the lowest number of items had a conditional reliability of 0.74 in CTT and of 0.86 in IRT. The exam with the highest number of items had a conditional reliability of 0.86 in CTT and of 0.93 in IRT.

### Medical school

We included data from three different medical schools in the study. Regarding conditional reliability at the cut score, we found neither a significant influence of the medical school (*B* = 0.020,* β* = 0.078, *p* = 0.37) nor a significant interaction with the used theory (*B* = −0.007, *β* = −0.032, *p* = 0.72).

### Percentage of MTF items

The percentage of MTF items ranged from 18.97% to 53.33% (mean = 30.60%). Regarding conditional reliability at the cut score, we found neither a significant influence of the percentage of MTF items (*B* = 0.471, *β* = 0.130, *p* = 0.09) nor a significant interaction with the used theory (*B* = −0.197, *β* = −0.074, *p* = 0.33).

## Discussion

In this study, we compared estimates of conditional reliability of 32 Swiss high-stakes medical exams in both Classical Test Theory (CTT) and Item Response Theory (IRT), with a special focus on the cut score and factors influencing the conditional reliability at the cut score. The first research question was whether previous findings regarding the areas with high and low precision in CTT and IRT can be replicated. As anticipated, we found that conditional reliability behaves in an inverse manner in the two theories. The second research question focused on how the conditional reliability at the cut score compares between the two theories. At the cut score, IRT showed higher conditional reliability compared with CTT, and the difference was statistically significant. Third, we analyzed whether the range of examinees’ performance, year of study and number of items influence conditional reliability in the two theories. We found that conditional reliability dropped as a function of the observed range of examinees’ scores and number of items in both IRT and CTT. The range of scores and year of study were highly correlated (*r* = −0.78). This decrease in the magnitude of the estimates was more pronounced in CTT. The medical schools and the amount of MTF items did not influence the results.

### Conditional reliability

As expected, we found differences between conditional reliability as estimated in CTT and IRT. Across exams, conditional reliability was at its maximum for the very high and the very low scores in CTT, whereas in IRT, conditional reliability was at its minimum for the very high scores.

In contrast to previous findings [12], we did not find extremely low reliability (i.e. <0.70) for the very low scores in IRT. This might be due to the restricted range of examinees’ scores, as all candidates were well prepared with a small percentage failing the exam and no examinees receiving zero points. Furthermore, measurement precision in IRT is dependent on the characteristics of the items included in the test. All exams in this study included a number of easy items in the exams which provide information (and thereby measurement precision) at the lower end of the ability continuum. Similar to Raju et al. [12], we found comparably low conditional reliability in IRT for the very high scores.

### Conditional reliability at the cut score

At the cut score, estimates of conditional reliability were higher in IRT compared with CTT, a difference that was statistically significant. Indeed, in IRT, reliability at the cut score was above 0.8 for 97% of the exams, while this was only the case for 30% of the exams when a CTT framework was employed. This result can be expected, since cut scores lay, on average, at a percentage-correct score of 56.7%. As delineated above, we observed the highest estimates of conditional reliability for IRT in exactly this range of test scores. This means that depending on the theory applied, rather different conclusions might be drawn on whether a sufficient level of measurement precision for making defensible pass-fail decisions has been reached. This finding might also have relevant practical implications, which will be addressed below.

### Influencing variables

With regard to influencing variables, we analyzed the range of examinees’ performance, year of study and number of items. We found that range of examinees performance and year of study were correlated ( *r* = 0.78), which demonstrates that cohorts indeed become more homogeneous as they progress through their studies. The smaller the range of examinees’ performance, the smaller the measurement precision at the cut score. The effect was more pronounced in CTT. This finding is in line with the literature considering estimates in IRT as independent of characteristics of the sample, whereas in CTT estimates, sample characteristics affect test statistics [16]. In CTT, conditional reliability at the cut score fell as low as 0.56 for very homogeneous groups. The second analyzed variable was the number of items, which also showed a significant influence on conditional reliability at the cut score. In both theories, a higher number of items led to higher conditional reliability at the cut score.

We included the medical school and the percentage of MTF items as control variables. These two variables did not affect the results. This shows that results are comparable in the three different schools and thereby they might also be transferable to other medical schools. The included exams consisted of both Type A and MTF items. MTF items are not the most commonly used type of items. We could show that the percentage of MTF items included did not influence the results. However, the amount of MTF items ranged between 18.97% and 53.33%. None of the included exams consisted only of Type A items. However, results regarding the distribution of conditional reliability were similar to those of Raju et al. [12] who used ‘dichotomously scored multiple choice items’. Therefore, we assume that results would be similar when using exams consisting of Type A items only. However, further research on this topic is needed.

### Strengths

To our knowledge, this is the first study to analyze conditional reliability in medical education assessment as well as potential influencing factors. Moreover, the study included a large sample of high-stakes medical education assessments with content-based cut scores and high-quality control and compared these aspects in two relevant psychometric theories. The sample included exams conducted at three different Swiss medical schools and represented all years of study.

### Limitations

The study included 32 high-stakes medical education exams. As all of these exams were end-of-term assessments with the aim to establish minimum competency, the assessments had similar characteristics. All cut scores were established in a content-based manner and ranged around 55%. All exams included large numbers of items. The results might differ for exams with small samples or different cut scores.

### Practical implications

Discussions about which theory to use in medical education assessment are still ongoing. Various studies comparing the practical implications of IRT and CTT found that many indices such as item difficulty, discrimination, global reliability and estimates of examinees’ ability are highly correlated [14, 31–34]. In this study, however, we demonstrated that regarding the concept of measurement precision, there is a noteworthy difference between IRT and CTT in terms of estimates of conditional reliability at the cut score. In addition, our results highlight that conditional reliability in IRT is more consistent across exams than in CTT. In particular, estimates based on IRT were less affected by decreasing between-person differences.

The finding that IRT and CTT lead to rather different estimates of conditional reliability at the cut score raises the question of which theory should be used under which conditions. While a thorough discussion of this topic is beyond the scope of the present paper, we argue that choosing a psychometric approach merely based on which provides higher estimates would be a dubious practice. However, we believe that IRT seems to provide a number of important features that do not easily translate into CTT. We will briefly discuss three noteworthy features of IRT below.

First, an intriguing feature of IRT is that it readily provides the basis for criterion-referenced interpretations of test scores; because both items and persons are explicitly linked to each other, the likelihood of answering an item correctly is a direct function of characteristics of the item and the examinee’s ability [14, 35]. As the aim of most exams in medical education within competency-based assessment is to ensure minimal ability, a criterion-based standard setting is commonly used [2]. Here, IRT offers a good fit for medical education assessments. Second, from a more technical perspective, IRT can be used for analyzing categorical data, which constitute the most common type of data in medical education assessment as items are mostly answered either correctly or incorrectly [13]. Third, from a conceptual point of view, IRT might be a more adequate fit for modeling the response process in typical clinical scenarios, since it conceives of the relation between ability and success on an item as an inherently stochastic process. This is an important conceptual feature, since more recent accounts for understanding the process of diagnostic inference and decision-making argue for the ‘probabilistic nature of diagnostic inference’ [36] and describe the physician as being situated in a probabilistic environment. If such a probabilistic environment can legitimately be assumed, methods developed within IRT may theoretically be an appropriate fit to model the process of responding to tasks and items in assessments in medical education. While the discussion on how and why to employ a specific psychometric framework warrants debate and should be looked at in more detail, we nevertheless believe that there are a number of reasonable arguments for opting for an IRT framework for typical medical education assessments, where minimal competency is crucial and criterion standard-setting is applied. Using IRT and thus conditional reliability in IRT to ensure measurement precision of pass-fail decisions may have practical implications for quality assurance and assessment design. As shown in our study, the number of items influences conditional reliability at the cut score, and even exams with a small number of items showed high conditional reliability (<0.8) in IRT. These findings indicate that using the concept of conditional reliability in IRT could inform exam design, for example by allowing for a smaller number of items if this is possible according to the blueprint. In terms of quality assurance, tests could be designed mainly comprising items that offer relevant information at the cut score. Thus, conditional reliability at the cut score could be increased and the overall number of items could be reduced.

## Conclusion

In this study, we compared conditional reliability estimates as calculated in Classical Test Theory (CTT) and Item Response Theory (IRT) with a special focus on the cut score. We showed that depending on the theory used, conditional reliability shows inverse distributions, and opposing conclusions about the measurement precision at the cut score. As the use of IRT seems to be more appropriate within competency-based education employing the criterion-oriented standard setting, these findings might have practical implications for the design and quality assurance of medical education assessments.
